# Innate Immune Pathways Associated with Lung Radioprotection by Soy Isoflavones

**DOI:** 10.3389/fonc.2017.00007

**Published:** 2017-01-23

**Authors:** Lisa M. Abernathy, Matthew D. Fountain, Michael C. Joiner, Gilda G. Hillman

**Affiliations:** ^1^Department of Oncology, Division of Radiation Oncology, Wayne State University School of Medicine, Detroit, MI, USA; ^2^Department of Immunology and Microbiology, Wayne State University School of Medicine, Detroit, MI, USA; ^3^Department of Microbiology and Immunology, Indiana University School of Medicine at Notre Dame, South Bend, IN, USA

**Keywords:** radiation, soy isoflavones, lung inflammation, MDSCs, arginase-1

## Abstract

**Introduction:**

Radiation therapy for lung cancer causes pneumonitis and fibrosis. Soy isoflavones protect against radiation-induced lung injury, but the mediators of radioprotection remain unclear. We investigated the effect of radiation on myeloid-derived suppressor cells (MDSCs) in the lung and their modulation by soy isoflavones for a potential role in protection from radiation-induced lung injury.

**Methods:**

BALB/c mice (5–6 weeks old) received a single 10 Gy dose of thoracic irradiation and soy isoflavones were orally administrated daily before and after radiation at 1 mg/day. Arginase-1 (Arg-1) and nuclear factor κB (NF-κB) p65 were detected in lung tissue by western blot analysis and immunohistochemistry. Lung MDSC subsets and their Arg-1 expression were analyzed by flow cytometry. Cytokine levels in the lungs were measured by ELISA.

**Results:**

At 1 week after radiation, CD11b^+^ cells expressing Arg-1 were decreased by radiation in lung tissue yet maintained in the lungs treated with radiation and soy isoflavones. Arg-1 was predominantly expressed by CD11b^+^Ly6C^low^Ly6G^+^ granulocytic MDSCs (gr-MDSCs). Arg-1 expression in gr-MDSCs was reduced by radiation and preserved by supplementation with soy isoflavones. A persistent increase in Arg-1^+^ cells was observed in lung tissue treated with combined radiation and soy isoflavones at early and late time points, compared to radiation alone. The increase in Arg-1 expression mediated by soy isoflavones could be associated with the inhibition of radiation-induced activation of NF-κB and the control of pro-inflammatory cytokine production demonstrated in this study.

**Conclusion:**

A radioprotective mechanism of soy isoflavones may involve the promotion of Arg-1-expressing gr-MDSCs that could play a role in downregulation of inflammation and lung radioprotection.

## Introduction

Non-small cell lung cancer (NSCLC) represents 85% of lung cancers, and the treatment of advanced disease remains a major clinical challenge (1, 2). Unresectable, stage III NSCLC is diagnosed in 50,000 Americans annually and is currently treated by concurrent chemoradiation therapy with a 5-year survival rate of only 20% (1, 2). The overall dose of radiation is limited by lung tissue toxicity presenting as radiation pneumonitis in up to 30% of NSCLC patients, necessitating means to improve the therapeutic ratio (3, 4).

Radiation pneumonitis is caused by an early inflammatory process triggered by tissue damage to lung parenchyma, epithelial cells, vascular endothelial cells, and stroma. This process induces the expression of pro-inflammatory cytokines and chemokines, which drive the recruitment of inflammatory cells into the injured tissue (5–7). Infiltrating inflammatory cells contribute to cyclical cytokine cascades responsible for clinical pneumonitis and late fibrosis (8, 9).

We have previously demonstrated that soy isoflavones protect against radiation-induced pneumonitis and fibrosis in naïve mice and xenografted lung tumor-bearing mice (10–12). In naïve mice, soy isoflavones given preradiation and postradiation reduced radiation injury to skin and hair follicles, protected respiratory function, and decreased inflammatory infiltrates and fibrosis (12). Significant decreases in pneumonitis, vascular damage, and fibrosis were also observed in lung tumor-bearing mice treated with soy isoflavones and radiation (10, 11). Furthermore, soy isoflavones differentially affected tumor versus lung tissue by radiosensitizing tumor nodules while radioprotecting normal lung tissue (11). We also recently reported that soy isoflavones reduced the extent of esophagitis caused by radiation within esophageal submucosal tissue layers (13). These findings suggest that soy isoflavones act in preventing inflammatory events in normal tissues within the field of thoracic irradiation. Recent efforts to determine modulation of inflammation by soy isoflavones revealed inhibition of the persistent infiltration and activation of macrophages and neutrophils at late time points of 12 and 18 weeks caused by radiation in the lungs (14). However, early immunosuppressive mediators of radioprotection by soy isoflavones remain to be identified.

To investigate the early events associated with soy isoflavone radioprotection, we have now studied immunosuppressive cells and molecules that could control the extent of inflammatory processes induced by radiation. Myeloid-derived suppressor cells (MDSCs) have been found to regulate inflammation induced by injury, pathogens, inflammatory diseases, and cancer (15, 16). MDSCs are a heterogeneous population of early myeloid progenitors, immature granulocytes, macrophages, and dendritic cells at different stages of differentiation (15, 17). Murine MDSCs express high levels of the CD11b myeloid lineage marker and the Gr-1 granulocytic marker. Gr-1 refers to two cell membrane molecules, Ly6C and Ly6G, and murine MDSCs are further classified into two subtypes, monocytic and granulocytic, according to their relative expression level. Monocytic MDSCs (mo-MDSCs) express high levels of Ly6C and low or no expression of Ly6G marker; granulocytic MDSCs (gr-MDSCs) express low levels of Ly6C and high levels of Ly6G (15, 17). MDSCs act by suppressing the function of lymphocytes including T cells, natural killer cells, and macrophages. Their immunosuppressive action is primarily mediated by affecting the metabolism of l-arginine, a common catalyzing substrate of NOS and arginase (15, 17). Arginase is an enzyme of the urea cycle that converts l-arginine to urea and exists as two isoforms: arginase 1 (Arg-1) and arginase 2 (Arg-2) (18). Arg-1 is associated with chronic inflammatory diseases, such as asthma and inflammatory bowel disease (19, 20). Arg-1 contributes to immune deactivation and is involved in inflammatory responses to LPS and pathogens (21, 22). Arg-1 can also reduce the activity of reactive oxygen species (ROS). Arg-1 expression by MDSCs could interfere with ROS induced by radiation that are involved in activation of nuclear factor κB (NF-κB) and transcription of inflammatory cytokines and chemokines (15, 22). In the current study, we have investigated whether Arg-1 expression in MDSCs could play a role in the decreased inflammation caused by soy isoflavones in irradiated lungs.

The effect of soy isoflavones on the expression of Arg-1 by MDSCs subsets in lung tissues was evaluated at early and late time points after radiation. Molecular events associated with Arg-1 expression involving NF-κB and inflammatory cytokines were also studied. Our findings suggest that high levels of Arg-1 expression by gr-MDSCs in the lungs are decreased by radiation but protected by soy isoflavones. The protection of Arg-1-positive immunosuppressive MDSCs by soy isoflavones could be associated with inhibition of radiation-induced NF-κB and inflammatory cytokines as a part of the radioprotection mechanisms.

## Materials and Methods

### Mice

Female BALB/c mice (Harlan, Indianapolis, IN, USA) of 5–6 weeks old were housed and handled in animal facilities accredited by the Association for Assessment and Accreditation of Laboratory Animal Care. The animal protocol was approved by Wayne State University Institutional Animal Care and Use Committee.

### Soy Isoflavones

The soy isoflavone mixture G-4660 used is a pure extract of 98.16% isoflavones from soybeans consisting of 83.3% genistein, 14.6% daidzein, and 0.26% glycitein (manufactured by Organic Technologies and obtained from NIH). The soy isoflavone mixture was dissolved in dimethyl sulfoxide (DMSO) and mixed with sesame seed oil at a 1:20 ratio just before treatment to facilitate gavage and avoid irritation of the esophagus by DMSO (10–12).

### Lung Irradiation

Radiation was delivered to the whole lung of anesthetized mice positioned in jigs under a 6.4-mm lead shield with cut outs to permit selective lung irradiation, as previously detailed (11). Photon irradiation was performed at 10 Gy with a Siemens Stabilipan X-ray set operated at 250 kV, 15 mA with 1 mm copper filtration at a distance of 47.5 cm from the target (14). The dose rate was 101 cGy/min, and half value layer was 2 mm Cu.

### Experimental Design

Mice were pretreated with oral soy isoflavones each day for 3 days at a dose of 5 mg/day (250 mg/kg), followed by lung irradiation at 10 Gy. Soy treatment was continued for 5 more days at 5 mg/day, and then a lower soy dose of 1 mg/day was given daily 5 days a week for up to 4 weeks, as previously detailed (11). In control mice and mice receiving irradiation alone, mice were treated with DMSO/sesame seed oil vehicle.

### Analysis of MDSC Subsets and Intracellular Arg-1 by Flow Cytometry

After bronchoalveolar lavage, the lungs were processed for single-cell suspension, and cells were immunostained using CD45-APC, CD11b-PE, Ly6C-PE-Cy7, and Ly6G-PE-Cy5.5 fluorescent antibodies (Abs), as previously described (14). After surface antigen staining, cells were fixed with 4% paraformaldehyde for intracellular Arg-1 staining (BD Biosciences, San Jose, CA, USA) followed by anti-IgG1-FITC. Cells were analyzed by flow cytometry using a BD LSR II flow cytometer (BD Biosciences, San Jose, CA, USA), followed by analysis on FlowJo v10 software (Tree Star Inc., Ashland, OR, USA) (14).

### Western Blot Analysis

Protein lysates were prepared from the lungs using a gentleMACS tissue dissociator (Miltenyi Biotec, San Diego, CA, USA). Total lung protein extracts (90 µg) were loaded and separated on 10% sodium dodecyl sulfate polyacrylamide gel electrophoresis and transferred to Whatman membranes. Membranes were incubated with anti-Arg-1 and anti-NF-κB p65 Abs (eBioscience) followed by horseradish peroxidase-conjugated secondary Ab. Membranes were reprobed with anti-β-actin Ab as a loading control, visualized by SuperSignal West Pico Chemiluminescent Substrate (ThermoFischer Scientific, Waltham, MA, USA) and captured on a Fotodyne imaging system (Fotodyne Inc., Hartland, WI, USA) (14).

### Immunohistochemistry (IHC)

The lungs were intratracheally instilled with 10% buffered formalin and resected, embedded in paraffin, and sectioned. Sections were incubated with anti-Arg-1 Ab following by biotinylated secondary Abs. Staining was amplified with the avidin-biotin immunoperoxidase technique, and slides were examined using Nikon E800 microscope (Nikon Inc., Melville, NY, USA) (14).

### Cytokine Analysis by ELISA

Lung homogenates were prepared using a gentleMACS tissue dissociator (Miltenyi Biotec, San Diego, CA, USA). Lung homogenates were assayed for interleukin (IL)-6, tumor necrosis factor (TNF)-α, IL-1β, and interferon (IFN)-γ using the respective Ready-SET-GO ELISA kits (eBioscience, San Diego, CA, USA) according to manufacturer’s instructions.

### Statistical Analysis

Comparisons between means of two treatment groups were analyzed by two-tailed unpaired Student’s *t*-test. Data were reanalyzed using ANOVA followed by Tukey’s Multiple Comparison Test, and the results confirmed the *t*-test significance values. A *p* value of <0.05 was considered statistically significant.

## Results

### Arg-1 Expression in CD11b^+^ Myeloid Populations Is Sustained by Soy Isoflavones at 1 Week Postradiation

**Figure 1:**
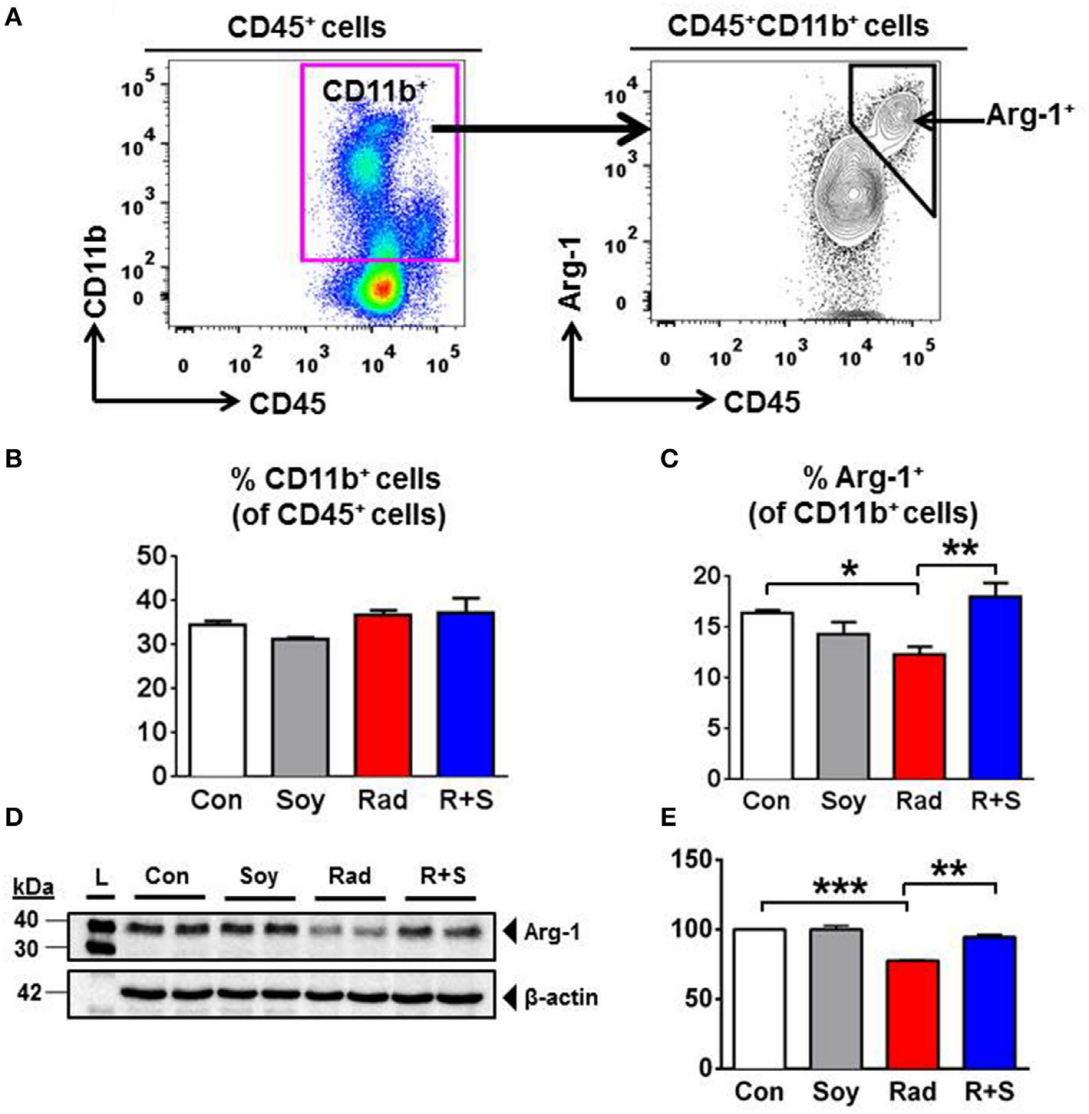
**Effect of soy isoflavones on intracellular arginase-1 (Arg-1) levels in CD11b^+^ leukocytes in the lungs at 1 week postradiation**. **(A–C)** Single-cell suspensions obtained from the lungs were analyzed by flow cytometry. **(A)** Gating strategy for CD11b^+^ and CD11b^+^Arg1^+^ cells. CD11b^+^ myeloid cells were gated within the CD45^+^ lung leukocytes. Intracellular Arg-1 cells were gated within the CD11b^+^ myeloid cells. **(B)** Analysis of CD45^+^CD11b^+^ leukocytes in the lungs from control (Con) and mice treated with soy (Soy), radiation (Rad), or radiation + soy (R + S). Percentages of CD11b^+^ cells within CD45^+^ cells are presented as mean ± SEM (*n* = 3 mice/group). **(C)** Intracellular Arg-1 expression in CD11b^+^ lung myeloid cells. Percentages of CD11b^+^Arg-1^+^ cells within CD45^+^ leukocytes are presented as mean ± SEM (*n* = 3 mice/group). *p < 0.05 for radiation compared to control, and **p < 0.01 or R + S compared to Rad alone. **(D,E)** Western blot analysis of Arg-1 on lung lysates obtained at 1 week after radiation. Radiation caused a significant decrease in Arg-1, but Arg-1 levels in R + S-treated lungs were comparable to those of Con or Soy lungs as confirmed in **(E)** by quantification of band intensities using ImageJ (NIH) densitometry analysis. The data are presented as mean ± SEM (*n* = 2 mice/group). ***p < 0.001 for radiation compared to control, and **p < 0.01 for R + S compared to radiation alone.

To address early molecular events modulated by soy isoflavones in the inflammatory response caused by radiation injury in the lungs, the expression of intracellular Arg-1 in lung CD11b^+^ myeloid cells was analyzed by flow cytometry at an early time point of 1 week after radiation (Figure 1A). Treatment of mice with lung irradiation and/or soy isoflavones did not affect the percentage of CD11b^+^ cells present in the lungs at 1 week postradiation, which represent about 35% of lung leukocytes (Figure 1B). However, radiation significantly decreased the percentage of Arg-1^+^ cells within the CD11b^+^ myeloid compartment in lungs compared to control (*p* < 0.05) (Figure 1C). Arg-1^+^ myeloid cells were sustained in lungs treated with radiation and soy isoflavones with a significant increase in CD11b^+^Arg-1^+^ cells compared to radiation (*p* < 0.01) (Figure 1C). These findings were confirmed by western blot analysis of Arg-1 overall expression in lung tissue lysates at 1 week after radiation, showing a significant decrease induced by radiation relative to control (*p* < 0.001) (Figures 1D,E). The addition of soy isoflavones resulted in higher Arg-1 levels compared to radiation alone (*p* < 0.01) (Figures 1D,E).

### Expression of Arg-1 in CD11b^+^ MDSCs Subsets in Lung Tissue

**Figure 2:**
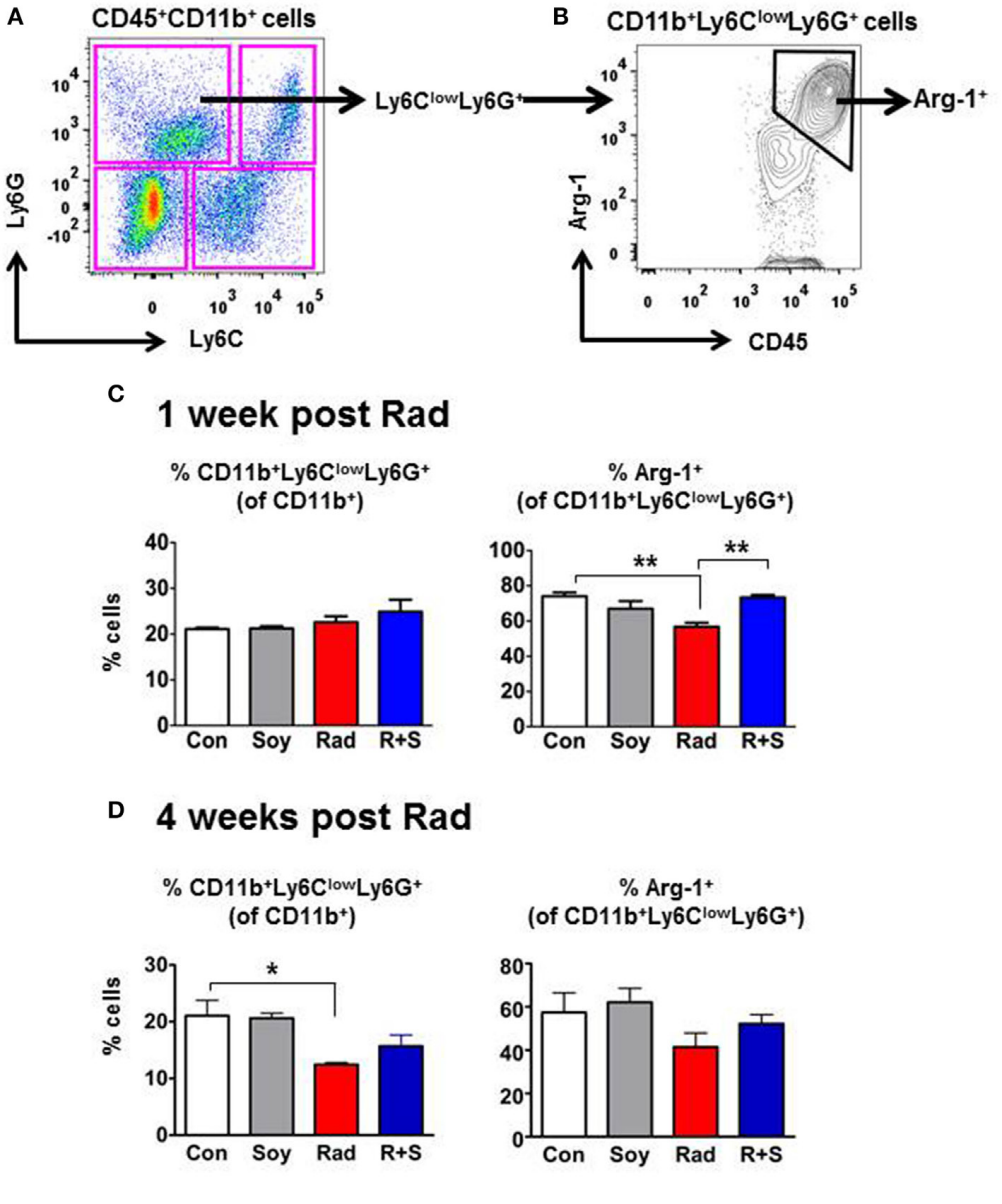
**Intracellular arginase-1 (Arg-1) expression of myeloid-derived suppressor cells (MDSCs) subsets at 1 and 4 weeks postradiation by flow cytometry analysis**. Single-cell suspensions isolated from the lungs were stained with anti-CD45, anti-CD11b, anti-Ly6C, and anti-Ly6G fluorescent antibodies to analyze CD11b^+^ myeloid subsets within CD45^+^ leukocyte populations by flow cytometry. **(A)** Gating of MDSCs subsets based on the expression of Ly6C and Ly6G within the CD45^+^CD11b^+^ cell population. **(B)** Gating of intracellular Arg-1^+^ within the CD11b^+^Ly6C^low^Ly6G^+^ granulocytic MDSC (arrows). **(C)** Analysis of CD11b^+^Ly6C^low^Ly6G^+^ granulocytic MDSC (gr-MDSC) subset and Arg1 expression at 1 week after radiation. Percentages of CD11b^+^Ly6C^low^Ly6G^+^ gr-MDSCs within the CD11b^+^ gated cells and percentages of Arg-1^+^ cells within CD11b^+^Ly6C^low^Ly6G^+^ cells are shown for lungs from control (Con) mice and mice treated with soy (Soy), radiation (Rad), or radiation + soy (R + S). **(D)** Analysis of CD11b^+^Ly6C^low^Ly6G^+^ gr-MDSC subset and their Arg1 expression at 4 weeks after radiation following treatment with Soy, Rad, and R + S. All data are presented as mean ± SEM (*n* = 3 mice/group). Significant data are labeled as **p < 0.01 or *p < 0.05 for radiation compared to Con or R + S compared to Rad alone.

To further interrogate the specific CD11b^+^ myeloid subsets in the lungs that are augmented by radiation and soy isoflavones at 1 week after radiation, intracellular expression of Arg-1 in MDSCs subsets was examined by flow cytometry. CD11b^+^ cell subsets were gated based on their differential expression of Ly6C and Ly6G to analyze mo-MDSCs (CD11b^+^Ly6C^+^Ly6G^−^) and granulocytic-MDSCs (CD11b^+^Ly6C^low^Ly6G^+^) (Figure 2A). The CD11b^+^Ly6C^low^Ly6G^+^ granulocytic-MDSCs (gr-MDSC) were of particular interest as this subset expressed the majority of Arg-1 in the lungs (70.07 ± 2.14% in control mice) (Figure 2B, arrow).

Further analysis focused on the CD11b^+^Ly6C^low^Ly6G^+^ gr-MDSC subset and showed that radiation and/or soy isoflavones treatment did not significantly affect their overall frequency (21–24% of CD11b^+^ cells) in the lung at 1 week postradiation compared to control (*p* > 0.05, Figure 2C). However, radiation significantly decreased the percentage of Arg-1-expressing cells within the CD11b^+^Ly6C^low^Ly6G^+^ gr-MDSCs subset from 74 ± 2.14% to 56.83 ± 2.20% compared to control (*p* < 0.01) (Figure 2C). Expression of Arg-1^+^ was significantly restored to 73.33 ± 1.41% in the CD11b^+^Ly6C^low^Ly6G^+^ gr-MDSC subset by treatment with radiation + soy isoflavones compared to radiation alone (*p* < 0.05), to levels comparable of control or soy-treated lungs (Figure 2C). Analysis of the gr-MDSC subset at a later time point of 4 weeks after radiation showed a significant decrease in this subset caused by radiation compared to control (*p* < 0.05) (Figure 2D). A trend to decreased Arg-1 expression in gr-MDSC subset was also observed by radiation but was not significant compared to control, which could reflect fluctuations in response to radiation at different time points. At 4 weeks after radiation, the percentages of CD11b^+^Ly6C^low^Ly6G^+^ gr-MDSC and the levels of Arg-1 expression were not altered in mice lungs treated with radiation + soy isoflavones and were not significantly different from control (*p* > 0.05) (Figure 2D).

Analysis of the CD11b^+^Ly6C^+^Ly6G^−^ mo-MDSC (Figure 2A, lower right quadrant) showed that this subset constitutes about 17–20% of CD11b^+^ cells. The percentages of mo-MDSC were not significantly changed by treatment with radiation, soy, or radiation + soy isoflavones compared to control mice at 1 or 4 weeks after radiation (*p* > 0.05). This subset showed a very low expression of Arg-1 of only 6% in contrast to the 70% levels in gr-MDSC.

### Increase in Arg-1^+^ Cells by Soy Isoflavones in Irradiated Lung Alveolar Septa

**Figure 3:**
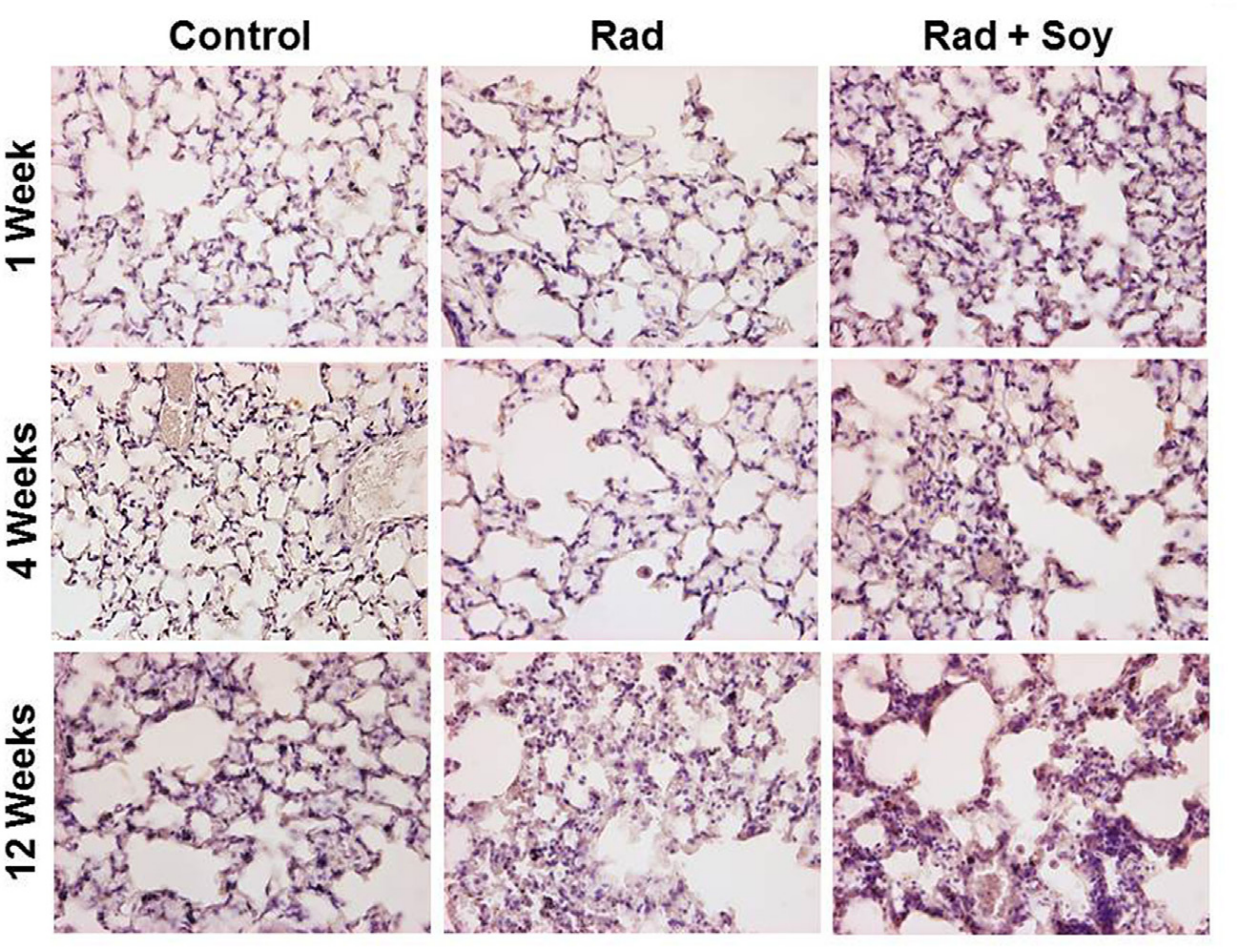
**Overall expression of arginase-1 (Arg-1) in the lung tissue treated with radiation and soy isoflavones (Rad + Soy) at 1, 4, and 12 weeks postradiation**. Lungs tissue sections obtained from control (Con) mice and mice treated with radiation (Rad) or Rad + Soy at 1, 4, and 12 weeks after radiation were stained for Arg-1 by immunohistochemistry. An increase in staining of Arg-1^+^ cells was seen in the lungs treated with Rad + Soy, compared to Rad, which persisted at 12 weeks. All magnifications are at 40×.

Overall expression of Arg-1 in cells in lung tissues was analyzed by IHC staining at early and late time points of 1, 4, and 12 weeks after radiation. At early and late time points after radiation, an increase in Arg-1^+^ cells was seen in alveolar septa from the lungs treated with radiation + soy isoflavones compared to control or radiation alone (Figure 3; Table 1). This persistent increase was observed evenly in cells in alveolar septa including large alveolar and interstitial macrophages as well as in smaller inflammatory cells. Consistent low levels of Arg-1^+^ cells were observed in the alveolar septa of lungs treated with radiation, compared to radiation + soy isoflavones (Figure 3; Table 1). Control lungs showed evenly distributed Arg-1^+^ cells (Figure 3; Table 1).

**Table 1:** **Histological scoring of arginase-1 (Arg-1)^+^ cells stained by immunohistochemistry in lungs**.

	Control	Radiation	Rad + Soy
1 week	+	+	+++
4 weeks	+	+	+++
12 weeks	++	++	++++

*Histological scoring for expression of Arg-1 shown in Figure 3 in the lung tissues treated with radiation and soy isoflavones at 1, 4, and 12 weeks after radiation using a scale from moderate (+), strong (++), very strong (+++), and heavy (++++)*.

### Soy Isoflavones Inhibit Radiation-Induced NF-κB p65 in Lung Tissue

**Figure 4:**
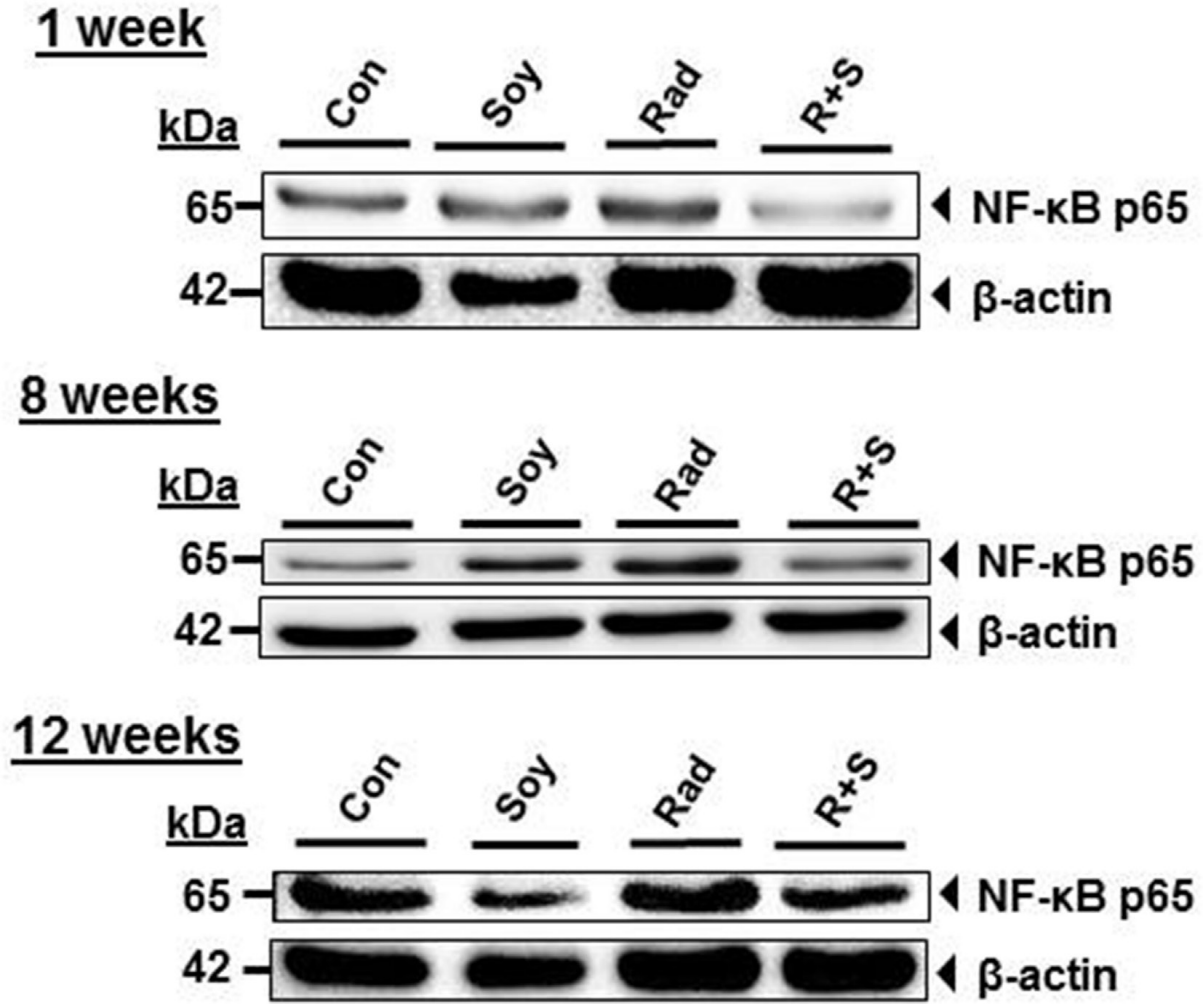
**Soy isoflavones inhibit radiation-induced increase of nuclear factor κB (NF-κB) p65 subunit levels in lung tissue**. Western blot analysis of NF-κB p65 using whole lung tissue lysates obtained from control (Con) mice and mice treated with soy isoflavones (Soy), radiation (Rad), or radiation + soy (R + S) at 1, 8, and 12 weeks after radiation. These data are representative of data reproduced in additional mice and in separate experiments.

Nuclear factor κB activation can be indirectly measured by the release of NF-κB p65 subunit from IκB. Therefore, the effect of radiation and soy isoflavones on the expression of NF-κB p65 subunit in lung lysate was analyzed by western blot. Radiation induced an increase in NF-κB p65 subunit protein levels at 1, 8, and 12 weeks postradiation compared to control and soy alone (Figure 4). The addition of soy isoflavones to radiation treatment consistently inhibited the expression of NF-κB p65 at each time point compared to the levels observed with radiation alone. These data were consistently reproduced in two to three separate experiments using either individual lungs from treated mice or lungs pooled from three mice per groups.

### Effect of Soy Isoflavones on Radiation-Induced Cytokines

**Figure 5:**
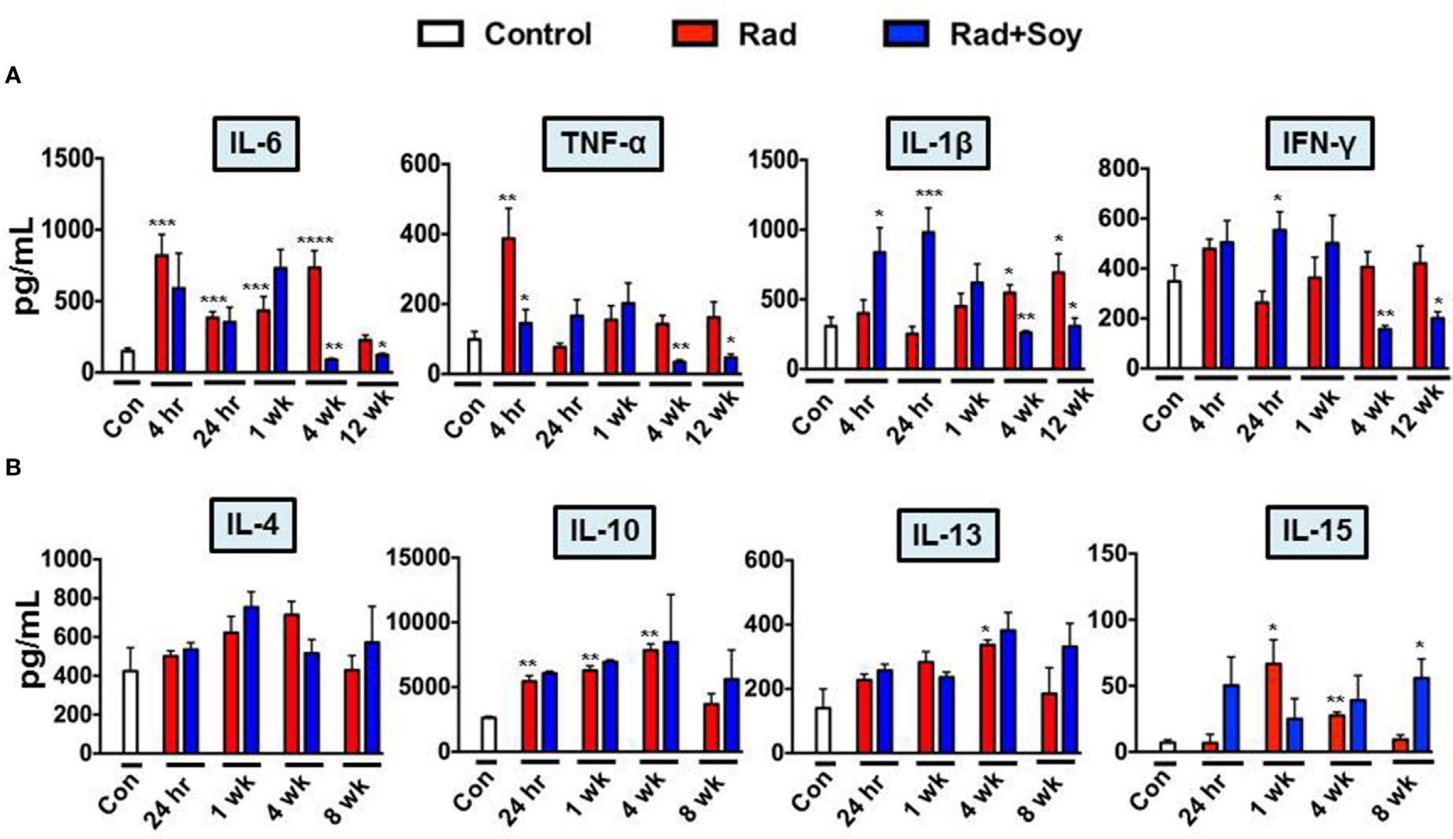
**Soy isoflavones modulation of cytokine production induced by radiation in lung tissue**. Cytokine levels were measured by ELISA in lung homogenates obtained from control mice (Con) and from treated mice at 4 h, 24 h, and 1, 4, 8, or 12 weeks following radiation (Rad) or radiation + soy isoflavones (Rad + Soy). **(A)** Kinetics of interleukin (IL)-6, tumor necrosis factor-α, IL-1β, and interferon-γ pro-inflammatory cytokines. **(B)** Kinetics of IL-4, IL-10, IL-13, and IL-15 anti-inflammatory cytokines. The data are presented as the mean ± SEM (*n* = 4–9 mice/group). *p < 0.05, **p < 0.01, and ***p < 0.001 when radiation is compared to control or when Rad + Soy is compared to Rad alone.

We showed that soy isoflavones inhibited expression of the NF-κB p65 subunit induced by radiation in lungs, and therefore, this could alter the transcription of inflammatory cytokines implicated in radiation-induced pulmonary injury (23, 24). IL-6, TNF-α, IL-1β, and IFN-γ pro-inflammatory cytokine levels were assessed at early time points of 4 h, 24 h, 1 week, and late time points of 4 and 12 weeks after radiation (Figure 5A). Compared to control, radiation induced a significant increase of IL-6 already seen at 4 h that remained high until 4 weeks after radiation (*p* < 0.001). Soy isoflavones significantly inhibited IL-6 induced by radiation at 4 weeks (*p* < 0.01) (Figure 5A). TNF-α levels in the lungs were initially increased by radiation early at 4 h (*p* < 0.01) but not by combined radiation and soy isoflavones (*p* < 0.01) (Figure 5A). At later time points after radiation, the levels of TNF-α remained low but with a further decrease mediated by soy isoflavones at 4 and 12 weeks (*p* < 0.01). Significant increases in IL-1β caused by radiation, at late time points of 4 and 12 weeks, were inhibited by soy isoflavones (*p* < 0.01) (Figure 5A). Radiation and soy isoflavones triggered a higher level of IL-1β early at 4 and 24 h (*p* < 0.01), but these levels dropped at 1 week. Fluctuations in IFN-γ levels were observed with higher levels by radiation and soy isoflavones at 24 h; however, by 4 and 12 weeks, the combined treatment caused significant decreases in IFN-γ compared to radiation (*p* < 0.01) (Figure 5A). Treatment of mice with soy isoflavones alone did not significantly affect the levels of these pro-inflammatory cytokines in the lungs (data not shown).

Studies of IL-4, IL-10, IL-13, and IL-15 anti-inflammatory cytokines showed increasing trends over time in the lungs treated by radiation alone or combined with soy isoflavones (Figure 5B). At a late time point of 8 weeks, a further increase was observed following radiation combined with soy isoflavones that was significant for IL-15 (*p* < 0.05) (Figure 5B). Treatment with soy isoflavones alone showed a trend toward increasing anti-inflammatory cytokines by 4 weeks of treatment (data not shown).

## Discussion

The pathology of lung injury following thoracic radiation is triggered by acute inflammation and promoted by pro-inflammatory cytokines that sets off a cascade of events akin to an aberrant wound healing response, with no resolution of inflammation but rather progression to fibrosis (6, 25, 26). We have previously reported that soy isoflavones inhibited the infiltration and activation of macrophages and neutrophils in irradiated lungs in naïve mice (14). However, soy isoflavones may also promote MDSCs to suppress inflammatory pathways induced by radiation injury. Expression of the enzyme Arg-1 by MDSC plays a key role in dampening inflammation (15, 17, 27). The effect of soy isoflavones on MDSC subsets in the lungs in association with Arg-1 expression was studied during the early phase of radiation-induced inflammation.

Flow cytometry analysis of CD11b^+^ myeloid cells revealed that the cells expressing Arg-1 were significantly decreased by radiation but preserved by soy isoflavones combined with radiation at an early time point of 1 week after radiation. Delineation of CD11b subsets based on Ly6C and Ly6G showed that intracellular Arg-1 was expressed in only about 6% of CD11b^+^Ly6C^+^Ly6G^−^ mo-MDSCs with no effect of soy and radiation. However, intracellular Arg-1 was predominant in CD11b^+^Ly6C^low^Ly6G^+^gr-MDSCs with 70% expression and was reduced by radiation but preserved by soy isoflavones. This trend was also observed at 4 weeks after radiation, suggesting a hypothetical role for gr-MDSCs expressing Arg-1 as an early event in the modulation of radiation-induced inflammation by soy isoflavones.

Histological studies confirmed that radiation caused an overall decrease in the expression of Arg-1 in cells of lung alveolar septa as early as 1 week after radiation, whereas treatment with radiation and soy isoflavones increased Arg-1 expression. This increase in Arg1^+^ cells observed in the lungs treated with radiation combined with soy isoflavones persisted for up to 12 weeks after radiation, suggesting modulation of molecular mediators of inflammation by soy isoflavones at both early and late time points after radiation. Expression of Arg-1 may be not only due to gr-MDSCs but also due to additional myeloid inflammatory cells. We have previously published that following radiation and soy treatment, alveolar macrophages in lung tissue express high levels of Arg-1 at a late time point of 18 weeks, which could be due to the promotion of M2 macrophages (14).

Elevated Arg-1 may suppress inflammatory pathways by competitively inhibiting inducible nitric oxide synthase (iNOS) and reducing ROS (22). Arg-1 expression by MDSCs could interfere with signaling events caused by ROS induced by radiation that are involved in NF-κB activation and transcription of pro-inflammatory cytokines (22, 28). Thoracic irradiation caused an increase in NF-κB p65 subunit levels as early as 1 week after radiation and was still observed by 12 weeks after radiation. The durable increase in NF-κB p65 subunit in irradiated lungs is reflective of continuous activation of molecular mediators involved in tissue inflammation triggered by radiation. However, addition of soy isoflavone treatment to radiation consistently inhibited NF-κB p65 levels at early and late time points after radiation.

Nuclear factor κB is essential for the transcription of inflammatory cytokines implicated in radiation-induced pneumonitis and fibrosis (25). We previously reported that soy isoflavones inhibit radiation-induced IL-6, TNF-α, IL-1β, and IFN-γ pro-inflammatory cytokines at late time points after radiation (11). These studies were further expanded to test pro-inflammatory and anti-inflammatory cytokines at early and late time points after radiation. Despite fluctuations in cytokine levels, soy isoflavones consistently inhibited IL-6, TNF-α, IL-1β, and IFN-γ pro-inflammatory cytokines at late time points of 4 to 12 weeks after radiation. Soy isoflavones caused a trend toward increasing levels of anti-inflammatory cytokines that was more significant with IL-15 but still noted with IL-4, IL-10, and IL-13 T_H_2 type cytokines. An anti-inflammatory role has been suggested for IL-15 cytokine to control T_H_1 immune responses in Crohn’s disease (29). The role of IL-15 in alleviation of radiation-induced inflammation by soy isoflavones warrants further investigation.

Our previous studies demonstrated that soy isoflavones inhibited the radiation-induced activation and infiltration of macrophages and neutrophils in lung tissues (14). Radiation induced a pro-inflammatory M1 macrophage phenotype, whereas addition of soy isoflavones caused a polarization to an anti-inflammatory M2 macrophage phenotype with increased levels of Arg-1 and decreased NOS2, which promote tissue repair (14). Our current study suggests that Arg-1-mediated resolution of inflammation could also occur during the early acute phase of radiation-induced inflammation. Interestingly, at late time points, soy isoflavones also protected immunoregulatory interstitial macrophages, which were decreased by radiation (14). Our findings are in agreement with other studies reporting that Arg-1 could attenuate the function of iNOS, inhibit NF-κB activation and inflammatory cytokines *in vitro*, and decrease macrophage infiltration and inflammation *in vivo* in a rabbit model of atherosclerosis (22). MDSCs played a critical role in the resolution of acute inflammation and tissue repair caused by spinal cord injury (30) and were found to modulate macrophage activation toward an immunosuppressive phenotype through Arg-1 (31). Whether early protection of Arg-1^+^ MDSC by soy isoflavones plays a role in altering molecular pathways involved in radiation-induced inflammation such as NF-κB activation and pro-inflammatory cytokine production remains to be established.

In summary, our preclinical study suggests that a radioprotective mechanism of soy isoflavones may involve, among other mechanisms, the promotion of gr-MDSCs that express Arg-1, which could play a role in downregulation of inflammation and ultimately lung radioprotection.

## Author Contributions

LA has conducted all the experiments described in this study, designed and performed the experiments, analyzed the data, and wrote the manuscript. MF has contributed his expertise in IHC studies, analysis of the data, and the writing of the manuscript. MJ was involved in the radiobiology design of the study. GH has directed and supervised all the studies and wrote the manuscript.

## Conflict of Interest Statement

The authors declare that the research was conducted in the absence of any commercial or financial relationships that could be construed as a potential conflict of interest. The reviewer ME and handling Editor declared their shared affiliation, and the handling Editor states that the process nevertheless met the standards of a fair and objective review.
